# Lung CD8+ T cells in COPD have increased expression of bacterial TLRs

**DOI:** 10.1186/1465-9921-14-13

**Published:** 2013-02-01

**Authors:** Christine M Freeman, Fernando J Martinez, MeiLan K Han, George R Washko,, Alexandra L McCubbrey, Stephen W Chensue, Douglas A Arenberg, Catherine A Meldrum, Lisa McCloskey, Jeffrey L Curtis

**Affiliations:** 1 Research Service, Department of Veterans Affairs Healthcare System, Ann Arbor, MI 48105, USA; 2 Division of Pulmonary & Critical Care Medicine, Department of Internal Medicine, University of Michigan Health System, Ann Arbor, MI 48109, USA; 3 Division of Pulmonary and Critical Care Medicine, Department of Medicine, Brigham & Women’s Hospital and Harvard University, Boston, MA, 02115, USA; 4 Graduate Program in Immunology, University of Michigan, Ann Arbor, MI, 48109, USA; 5 Pathology & Laboratory Medicine Service, VA Ann Arbor Healthcare System, Ann Arbor, MI, 48105, USA; 6 Medical Service, VA Ann Arbor Healthcare System, Ann Arbor, MI, 48105, USA; 7 Department of Pathology, University of Michigan Health System, Ann Arbor, MI, 48109, USA

**Keywords:** Chronic obstructive pulmonary disease, CD8+ T cells, Toll-like receptors, Lung

## Abstract

**Background:**

Toll-like receptors (TLRs) on T cells can modulate their responses, however, the extent and significance of TLR expression by lung T cells, NK cells, or NKT cells in chronic obstructive pulmonary disease (COPD) is unknown.

**Methods:**

Lung tissue collected from clinically-indicated resections *(n = 34)* was used either: (a) to compare the expression of TLR1, TLR2, TLR2/1, TLR3, TLR4, TLR5, TLR6 and TLR9 on lung CD8+ T cells, CD4+ T cells, NK cells and NKT cells from smokers with or without COPD; or (b) to isolate CD8+ T cells for culture with anti-CD3ε without or with various TLR ligands. We measured protein expression of IFN-γ, TNF-α, IL-13, perforin, granzyme A, granzyme B, soluble FasL, CCL2, CCL3, CCL4, CCL5, CCL11, and CXCL9 in supernatants.

**Results:**

All the lung subsets analyzed demonstrated low levels of specific TLR expression, but the percentage of CD8+ T cells expressing TLR1, TLR2, TLR4, TLR6 and TLR2/1 was significantly increased in COPD subjects relative to those without COPD. In contrast, from the same subjects, only TLR2/1 and TLR2 on lung CD4+ T cells and CD8+ NKT cells, respectively, showed a significant increase in COPD and there was no difference in TLR expression on lung CD56+ NK cells. Production of the Tc1 cytokines IFN-γ and TNF-α by lung CD8+ T cells were significantly increased via co-stimulation by Pam3CSK4, a specific TLR2/1 ligand, but not by other agonists. Furthermore, this increase in cytokine production was specific to lung CD8+ T cells from patients with COPD as compared to lung CD8+ T cells from smokers without COPD.

**Conclusions:**

These data suggest that as lung function worsens in COPD, the auto-aggressive behavior of lung CD8+ T cells could increase in response to microbial TLR ligands, specifically ligands against TLR2/1.

## Background

Chronic obstructive pulmonary disease (COPD), the 3^rd^ leading cause of death in the United States, is a chronic, debilitating disease with a rapidly increasing incidence [1]. Although COPD is recognized to have important systemic manifestations [2], its principal pathological changes occur in the lungs and include irreversible lung destruction, airways remodeling, mucus hypersecretion, and inflammatory cell infiltration [3]. This infiltration is characterized by increased numbers of macrophages, neutrophils, and CD8+ T cells in the lungs. CD8+ T cells have been implicated in COPD pathogenesis primarily because their numbers in lung parenchyma and small airways correlate inversely with forced expiratory volume in one second (FEV_1_) [4,5], the modality most frequently used to define COPD severity. We have previously shown that lung CD8+ T cell mRNA transcripts for IFN-γ, perforin, and granzyme B correlate inversely to FEV_1_% predicted [6,7]. Based on the ability of CD8+ T cells to kill cells that they recognize as infected or damaged, and to secrete cytokines that can recruit and activate additional inflammatory cells, CD8+ T cells have considerable potential to damage lung parenchyma. Nevertheless, despite support in murine models [8,9], there is no direct evidence that human lung CD8+ T cells mediate such damage in COPD and it is unclear what mechanisms drive this amplified CD8 response in the minority of smokers who develop COPD.

The function of lung CD4+ T cells, natural killer (NK) cells and natural killer T (NKT) cells in COPD has been less widely studied. There is evidence that numbers of CD4+ T cells and NK cells, but not of NK T cells, also increase in the lung as the disease progresses [3,10,11]. In peripheral blood, the cytotoxic effector function of NK and NKT cells appears to be impaired and their relative number to be reduced in COPD subjects [12,13]. However, the same investigators later demonstrated that NK and NKT cells collected from the sputum were both higher as a proportion of all leukocytes and more cytotoxic in COPD subjects than in healthy smokers or non-smoking healthy subjects [14]. A similar finding was also very recently reported for NK and NKT cells in the bronchoalveolar lavage fluid from individuals with COPD [15]. As NK and NKT cells can be a potent source of cytokines and cytotoxic molecules, an increase in their number or improper effector function could contribute to COPD pathology.

Recent data from both human and murine studies have revealed that Toll-like receptors (TLRs), originally described on innate immune cells, also modulate T cell responses. mRNA for TLR1, TLR2, TLR3, TLR4, TLR5, TLR7 and TLR9 has been detected in human peripheral blood T cells, although at greatly varying levels [16]. Engagement of TLRs on T cells lowered the activation threshold for co-stimulatory signals delivered by antigen-presenting cells [16-18]. TLR3 and TLR9 stimulation enhanced the survival of activated murine CD4+ T cells, without augmenting their proliferation [18]. IFN-γ production was increased by stimulation via TLR3 or TLR4 in human CD8+ T cells [19,20] and via TLR5 or TLR7/8 in human cells positive for CD45RO, which would consist predominately of memory T cells [16]. Evidence also suggests that TLR stimulation of CD8+ T cells enhances their cytotoxicity. Co-stimulating activated T cells with TLR2 ligands upregulated granzyme B secretion and cytotoxic activity [17]. Furthermore, TLR3 and TLR9 ligands induced fully efficient cytotoxic responses by murine CD8+ T cells, overriding the requirement for CD4+ T cell help [21]. In contrast, a different study found that engagement of TLR3 on human CD8+ T cells had no effect on their cytolytic activity [19]. Human peripheral blood NK cells reportedly express high levels of TLR3, TLR7, and TLR9 and stimulation with ligands led to an increase in IFN-γ production [22]. In a mouse model of COPD, NK cells from smoke-exposed mice produced more IFN-γ following stimulation with the ligands for TLR3, TLR7, and TLR9 than NK cells from mice exposed to filtered air [23].

By contrast, relatively little is know about the expression of TLRs by lung T cells, NK cells, or NKT cells in COPD. In endobronchial biopsies, TLR4 and TLR9 expression was increased on CD8+ T cells of subjects with COPD as compared to control subjects. Stimulation of peripheral blood CD8+ T cells with cigarette smoke condensate led to increases in TNF-α and IL-10, that were attenuated following TLR4 or TLR9 inhibition [24]. However, the role of other TLRs or specific TLR agonists was not explored.

Based on the ongoing colonization of the lower airways in COPD by microbial pathogens, we hypothesize that modulation from TLR signaling could contribute to the exaggerated pathogenic response of lung CD8+ T cells in COPD. To test this hypothesis, we used flow cytometry to analyze TLR expression on lung CD8+ T cells (CD3+ CD8+ CD56-), CD4+ T cells (CD3+ CD4+ CD56-), NK cells (CD3- CD56+ CD4- CD8-), CD4 NKT cells (CD3+ CD56+ CD4+ CD8-) and CD8 NKT cells (CD3+ CD56+ CD8+ CD4-). After observing increases in the TLR expression on CD8+ T cells from COPD subjects compared to subjects with normal pulmonary function, we assessed the functional relevance of this expression by stimulating isolated CD8+ T cells with TLR ligands and measuring production of cytokines, cytotoxic molecules, and chemokines. Our data show that human lung CD8+ T cells responded to TLR agonists, in particular the TLR2/1 agonist Pam3CSK4, by increasing production of cytokines and chemokines in response to TCR stimulation.

## Methods

### Specimens and patient population

Consented subjects undergoing clinically-indicated resections for pulmonary nodules, lung volume reduction surgery, or lung transplantation were recruited from the University of Michigan Health System and the VA Ann Arbor Healthcare System. Studies and consent procedures were approved by Institutional Review Boards. All subjects (*n* = 34) gave written consent preoperatively and underwent preoperative spirometry, prospectively collected medication history and clinical evaluation by a pulmonologist. Available non-contrast-enhanced CT scans (*n* = 13) were analyzed for percent emphysema using 3D Slicer software (http://www.airwayinspector.org) at a threshold of < −950 Hounsfield units. We categorized subjects using the 2008 classification of the Global Initiative for Chronic Obstructive Lung Disease (GOLD) [25]. Subjects (*n* = 14) with a smoking history of 10 pack years or greater, a ratio of forced expiratory volume in 1 second to forced vital capacity (FEV_1_/FVC) >0.70, normal spirometry, and no clinical diagnosis of COPD represent smoking controls (S). Subjects (*n* = 20) with a smoking history, defined as greater than 10 pack years, FEV_1_/FVC <0.7 and abnormal spirometry were considered to have COPD. Table 1 shows the male-to-female ratio, age, smoking history, FEV_1_% predicted, and% emphysema for both subject categories. Former smokers were defined as those subjects who had quit smoking for greater than 6 months.

**Table 1:** Summary of subject demographics, smoking histories, spirometry and emphysema

**Group**	**S**	**COPD**	***p*value**
Subjects, n	14	20	
Sex ratio, M/F	11/3	12/8	NS
Age, years (SD)	62 (9)	62 (11)	NS
Smoking, pack-years (SD)	60 (29)	62 (41)	NS
Smoking status (Active/Former)	6/8	5/15	NS
FEV_1_,% pred (SD)	91 (16)	40 (27)	<0.0001
Emphysema,% (SD)	12 (14)	36 (15)	0.02
Surgical indication (resection/LVRS/transplant)	14/0/0	8/5/7	0.001
ICS use (yes/no)	1/13	14/6	0.0004
Recent infection (yes/no)	0/14	2/18	NS

Data are presented as average (SD), except for sex and smoking status ratios. Former smokers were defined as those subjects that had quit smoking for more than 6 months. Subjects were considered to have a recent infection if they had taken a course of antibiotics in the past 6 weeks. S, smoker without COPD; M, male; F, female; LVRS, lung volume reduction surgery; ICS, inhaled corticosteroids; NS, not significant.

Studies and consent procedures were performed in accordance with the Declaration of Helsinki at the VA Ann Arbor Healthcare System and the University of Michigan Health System and were approved by the Institutional Review Board at each site (FWA 00000348 and FWA 00004969, respectively).

### Sample preparation and flow cytometric analysis

Under the supervision of a Pathologist, in cases of nodule resection, only parenchymal, non-neoplastic lung tissue remote from the nodules and lacking post-obstructive changes was collected. In the cases of explanted lungs, tissue from the distal region of the lung was collected. Lung specimens were immediately stored in fresh RPMI and kept at 4°C until processed. Lung sections weighing approximately 3 g were dispersed using a Waring blender without enzyme treatments, which we have previously shown produces single cell suspensions of high viability and functional capacity [6]. Importantly, not all types of experiments were performed on tissue from every subject, due to limitations in the size of some samples.

For flow cytometry, cells were filtered through a 40 μm strainer to remove debris and were resuspended in staining buffer (2% FBS in PBS). Cells were added in a volume of 100 μl to each flow tube. We used monoclonal antibodies against the following antigens (clones shown in parentheses): CD45 (HI30), CD3 (UCHT1), CD4 (OKT4), CD8 (OKT8), CD56 (MEM-188), TLR1 (GD2.F4), TLR2 (T2.5), TLR3 (TLR3.7), TLR4 (HTA125), TLR6 (hPer6), TLR9 (eB72-1665), (eBioscience, San Diego, CA), and TLR5 (85B152.5) (Abcam, Cambridge, MA). Appropriate isotype-matched controls were used in all experiments. Antibodies were conjugated to either fluorescein isothiocyanate (FITC), phycoerythrin (PE), phycoerythrin-cyanine 7 (PE-Cy7), allophycocyanin (APC), allophycocyanin-cyanine 7 (APC-Cy7), Pacific Blue, Alexa Fluor 700, or biotin, with the biotinylated antibodies developed using streptavidin-phycoerythrin-cyanine 5 (SA-PE-Cy5), as shown in Table 2.

**Table 2:** Antibody staining panel

**Fluorochrome**	**1**	**2**	**3**	**4**	**5**	**6**
FITC	M IgG2a	TLR5	CD45	CD45	CD45	CD45
PE	IC:M IgG1	IC:TLR3	M IgG1	TLR1	IC:R IgG2a	IC:TLR9
Biotin (PE-Cy5)			R IgG2a	TLR6		
PE-Cy7	CD45	CD45	M IgG1	TLR2	M IgG2a	TLR4
APC	M IgG2a	CD56	M IgG2a	CD56	M IgG2a	CD56
APC-Cy7	M IgG1	CD3	M IgG1	CD3	M IgG1	CD3
Pacific Blue	M IgG2a	CD8	M IgG2a	CD8	M IgG2a	CD8
Alexa Fluor 700	M IgG2b	CD4	M IgG2b	CD4	M IgG2b	CD4
Aqua (AmCyan)	Live/Dead	Live/Dead	Live/Dead	Live/Dead	Live/Dead	Live/Dead

M, mouse; R, rat; IC, intra-cellular.

To evaluate cell viability by flow cytometry, we used LIVE/DEAD^®^ Fixable Dead Cell Stain Kit (Life Technologies) in Aqua. This reactive dye permeates the membranes of necrotic cells, resulting in positive fluorescent staining. This procedure is also compatible with intracellular staining. Cells were first incubated in the dark with the LIVE/DEAD^®^ Fixable dye for 30 minutes, washed, and then incubated with primary antibodies and secondary reagents for 25 minutes each at room temperature, with washing between incubations. We measured intracellular expression of the endosomal receptors TLR3 and TLR9, after cell fixation and permeabilization. Cells were analyzed on an LSR II flow cytometer (BD Bioscience, San Jose, CA) equipped with 488 nm blue, 405 nm violet, 633 nm red lasers. Data were collected using FACS Diva software with automatic compensation, and were analyzed using FlowJo software (Tree Star, Inc., Ashland, OR). A minimum of 10,000 viable (cells that are negative for the Aqua Live/Dead fluorescence) CD45+ events were collected per sample.

### In vitro stimulation

In separate experiments, CD8+ CD56- T cells were isolated from lung tissue by sequentially incubating the single-cell suspension with magnetic beads (Miltenyi Biotec, Auburn, CA). Samples were first incubated with CD56 magnetic beads for 15 minutes at 4°C and positively selected using MACS LS columns (Miltenyi Biotec). The remaining CD56 negative cells, which no longer contained NK cells, were then incubated with CD8 magnetic beads and positively selected using MACS LS columns. In these experiments, viability was determined by Trypan blue exclusion. CD8+ cells were cultured in 96-well flat-bottom plates at a density of 50,000 viable cells per 200 μl lymphocyte culture media (10% FBS, 1 mM sodium pyruvate, 0.5 mM 2-Mercaptoethanol, 1 mM HEPES, 100 u/ml penicillin, 100 u/ml streptomycin, 0.292 mg/ml L-Glutamine in RPMI). Cells were stimulated with media alone, with plate-bound anti-CD3ε (5 μg/ml) alone, or with anti-CD3ε plus each of the following TLR ligands: Pam3CSK4 (1 μg/ml), Heat-killed *Listeria monocytogenes* (HKLM; 10^8^ organisms/ml), Poly(I:C) (10 μg/ml), LPS *E. coli* K12 (1 μg/ml), Flagellin *Salmonella typhimurium* (1 μg/ml), FSL-1 (1 μg/ml), Imiquimod (1 μg/ml), ssRNA 40 (1 μg/ml), or ODN 2006, type B (5 μM) (all agonists from InvivoGen, San Diego, CA) Cells were cultured at 37°C and 5% CO_2_ for 48 hours, at which point supernatants were collected for analysis.

### Protein analysis of culture supernatants

Cell culture supernatants were collected and stored at −20°C until analyzed. Using the Luminex 200 system (Luminex Corporation, Austin, TX), protein levels for IFN-γ, TNF-α, IL-13, perforin, granzyme A, granzyme B, soluble Fas ligand, (Biolegend, San Diego, CA) and CCL2, CCL3, CCL4, CCL5, CCL11, and CXCL9 (Invitrogen, Carlsbad, CA) were determined according to manufacturer’s instructions.

### Statistics

Statistical analysis was performed using SAS 9.1 statistical software (SAS Institute Inc., Cary, NC) and GraphPad Prism (GraphPad Software, Inc., La Jolla, CA). The Mann–Whitney t-test was used to compare TLR expression between subjects with COPD and subjects with normal pulmonary function. Kruskal-Wallis tests were used to look for significant differences between TLR-ligand treatment groups. Nonparametric (Spearman) correlation analysis was used to determine the correlation coefficient, *r*_*S*_. Both linear and log transformed outcomes were considered. Multivariate regression examined the statistically significant relationships, adjusting for age and smoking exposure (pack years). A two-tailed *p* value of < 0.05 was considered to indicate significance.

## Results

### Identification of CD8+ T cell, CD4+ T cell, and NK cell subsets in human lung tissue

**Figure 1:**
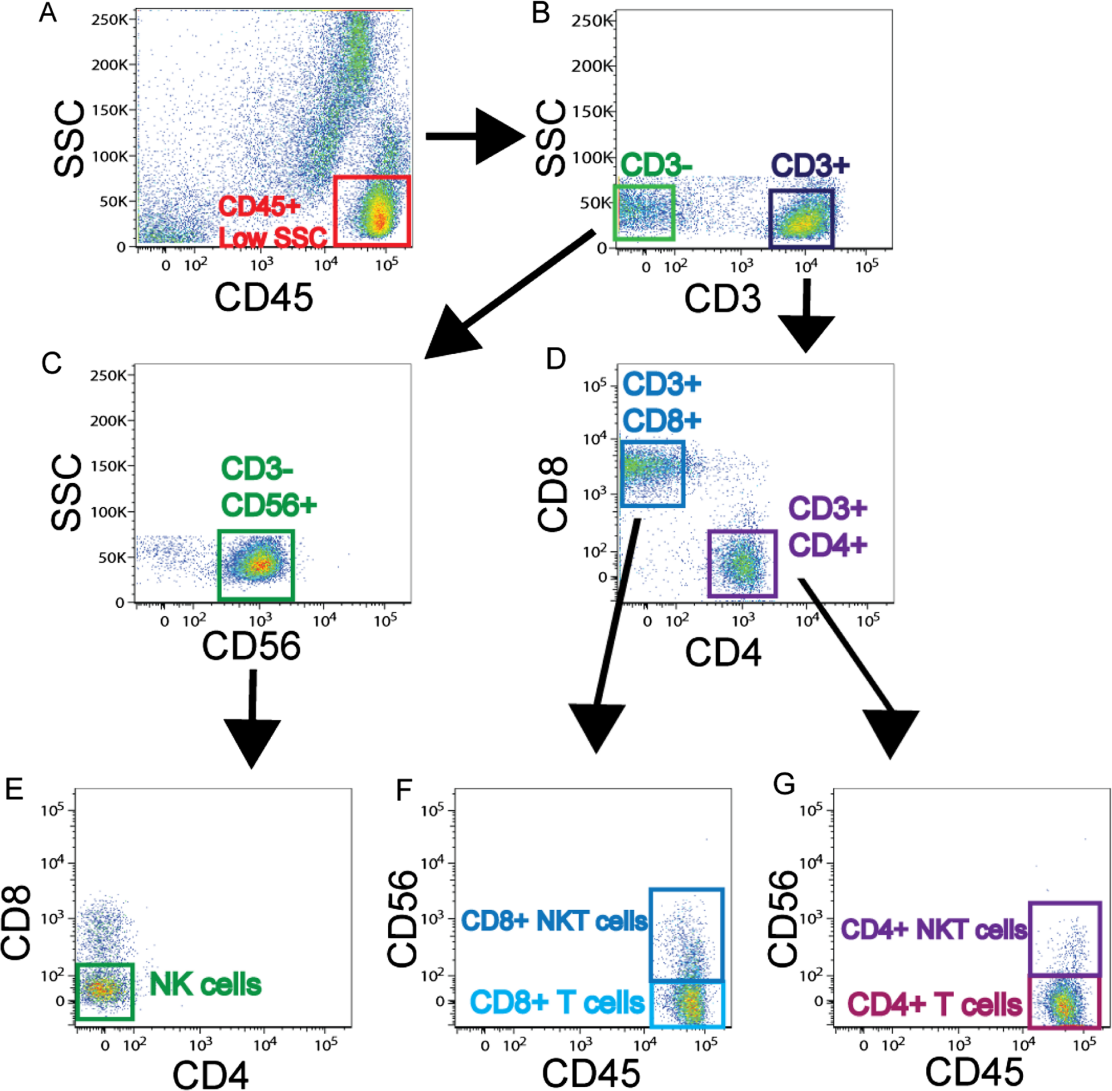
**Gating strategy used to identify T cells, NK cells, and NKT cells isolated from human lung tissue.** Lung tissue was dispersed and stained with CD45, CD3, CD8, CD4, and CD56 monoclonal antibodies to identify various cell subsets. (**A**) Viable CD45+ cells with a low side scatter profile were selected; (**B**) cells were further divided into CD3- and CD3+ fractions; (**C**) CD56+ cells were selected from the CD3- fraction and (**E**) NK cells were selected by excluding CD4 and CD8. (**D**) CD8+ and CD4+ cells were identified and (**F**) CD56+ was used to separate CD8+ NKT cells from CD8+ T cells and (**G**) CD4+ NKT cells from CD4+ T cells. Appropriate isotype-matched controls for all antibodies were used to determine positive staining.

To determine whether lung T cells, NK cells and NK T cells express TLRs, we used 9-color flow cytometry to analyze expression of TLR1, TLR2/1, TLR2, TLR3, TLR4, TLR5, TLR6 and TLR9 on unpurified lung leukocytes from 28 subjects. We assessed both surface and intra-cellular expression of TLR3 and TLR9. We identified five different lymphocyte populations, including CD8+ T cells, CD4+ T cells, NK cells, CD8+ NKT cells, and CD4+ NKT cells. To identify these populations, we first gated on CD45+ low side scatter cells (Figure 1A) and then selected cells that were either CD3+ or CD3- (Figure 1B). NK cells were defined as CD3- CD56+ CD4- CD8- cells (Figure 1C, E). CD8+ T cells were defined as being CD3+ CD8+ CD56-, while CD8+ NKT cells were CD3+ CD8+ CD56+ (Figure 1D, F). Similarly, CD4+ T cells were defined as CD3+ CD4+ CD56- cells and CD4+ NKT cells were CD3+ CD4+ CD56+ (Figure 1D, G). We saw no difference in the frequency of any of the cell types when comparing healthy smokers to COPD subjects (data not shown). On average, the frequency as a percent of CD45+ cells was 7.6% for CD8+ T cells, 8.8% for CD4+ T cells, 5.0% for NK cells, 1.3% for CD8+ NKT cells, and 0.6% for CD4+ NKT cells.

### Increased percentage of lung CD8+ T cells expressing TLRs in COPD and emphysema

**Figure 2:**
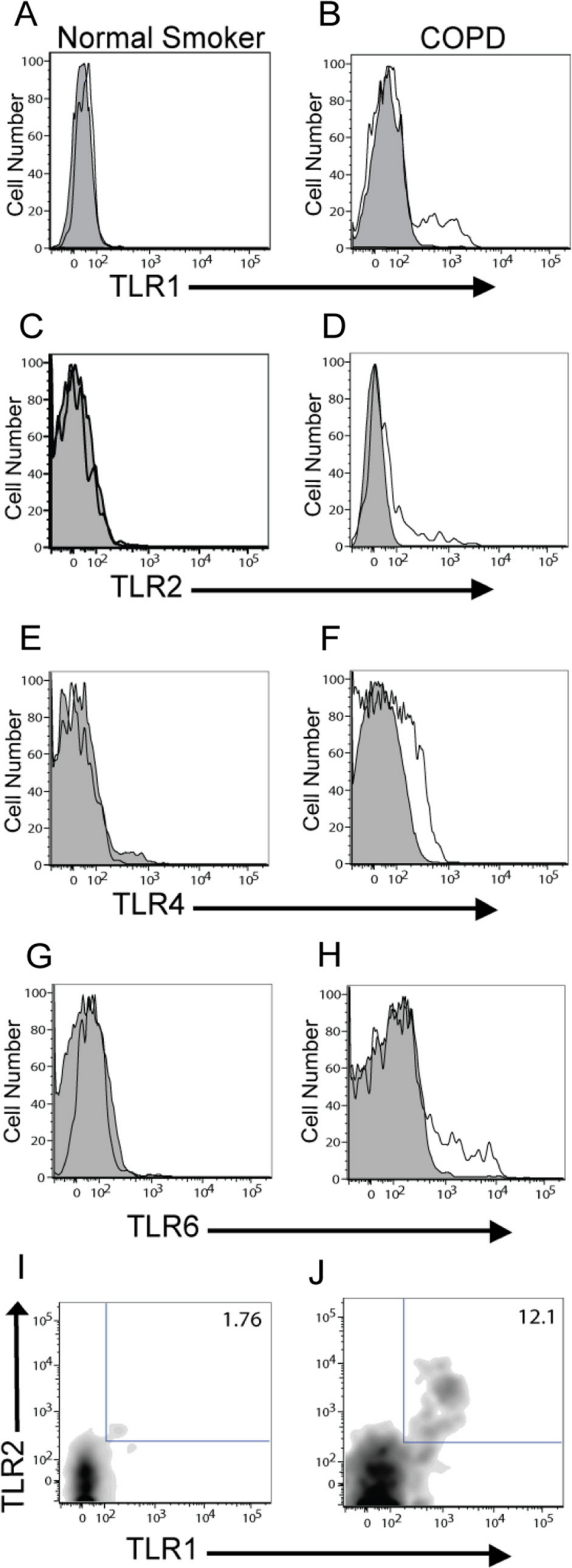
**Representative histograms demonstrating an increase in TLR expression on lung CD8+ T cells in COPD.** CD8+ CD56- cells from human lung tissue were identified as described in the legend to Figure 1 and examined for TLR expression. Left panels are representative histograms (**A, C, E, G**) or density plot (**I**) from smokers without COPD and right panels are representative histograms (**B, D, F, H**) or density plot (**J**) from subjects with COPD. Expression of (**A, B**) TLR1, (**C, D**) TLR2, (**E, F**) TLR4, (**G, H**) TLR6, and (**I, J**) TLR2/1 are shown. Shaded profile, isotype staining; open profiles, antibody-specific staining.

**Figure 3:**
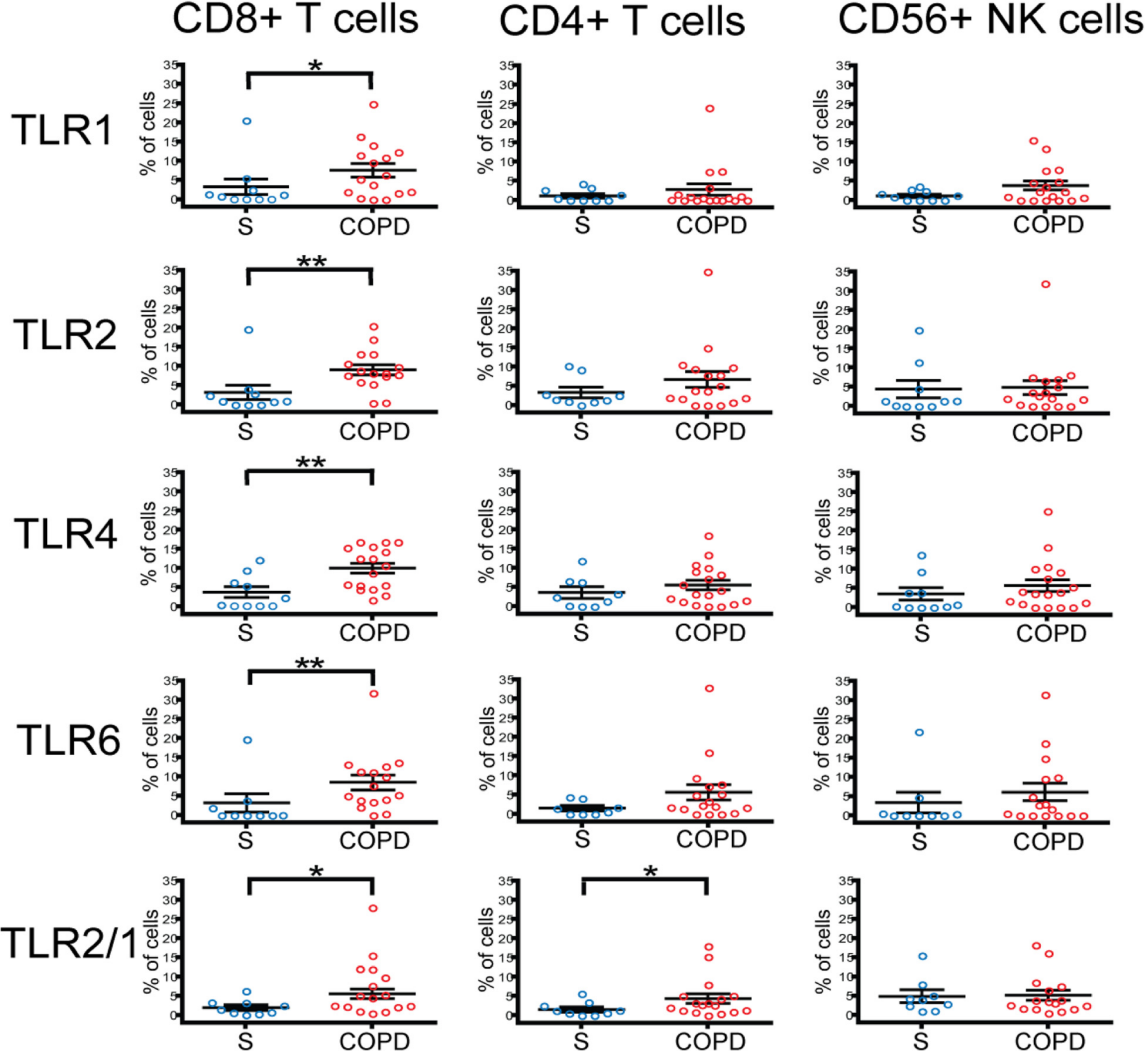
**Percentage of CD8+ T cells expressing bacterial TLRs is increased in COPD subjects.** Lung tissue was dispersed and stained with monoclonal antibodies to measure TLR expression on CD8+ T cells (left panels), CD4+ T cells (middle panels), or CD56+ NK cells (right panels) from smokers without COPD (S; blue circles; *n* = 10) and with COPD (COPD; red circles; *n* = 18). The vertical axis shows the percentage of cells expressing a given TLR. Open circles represent individual patients. Lines represent the mean ± SEM. The Mann Whitney t-test was used to determine significant differences between groups. *, p < 0.05, **, p < 0.01.

After gating on our cell population of interest, we used a 5% probability contour plot to select positive events, as distinguished from negative events by the isotype control. With this strategy, we were able to determine the percentage of CD8+ T cells, CD4+ T cells, NK cells and NK T cells that expressed a given TLR. Analysis of the TLR expression on 28 subjects (*n* = 10 subjects with normal pulmonary function, *n* = 18 subjects with COPD) revealed that the percentage of lung CD8+ T cells expressing TLR1, TLR2, TLR4, TLR6, and TLR2/1 was significantly increased in subjects with COPD compared to the subjects without COPD (Figure 2 and Figure 3, left panel). Lung CD8+ T cells also expressed TLR3, TLR5, and TLR9 (~10% of cells), but the percentage of positive cells did not show a significant difference between subject groups (data not shown). Interestingly, in contrast to the CD8+ T cell findings, although CD4+ T cells, CD56+ NK cells and NKT cells were analyzed simultaneously in the same sample, only the expression of TLR2/1 on the lung CD4+ T cells and the expression of TLR2 on the CD8+ NKT cells was significantly increased in the subjects with COPD (Figure 3, middle and right panels; data not shown for NKT cells). As shown in the subject demographics (Table 1), healthy smokers and subjects with COPD showed significant differences in their clinical indication for surgery and their inhaled corticosteroid (ICS) use. To ensure that the presence of lung cancer or ICS use were not responsible for the difference in TLR expression between healthy smokers and COPD subjects, we analyzed these parameters in the COPD cohort. We saw no difference in TLR expression when the subjects with COPD were stratified by surgical indication or ICS use, suggesting that these variables are not contributing to the difference in TLR expression.

**Figure 4:**
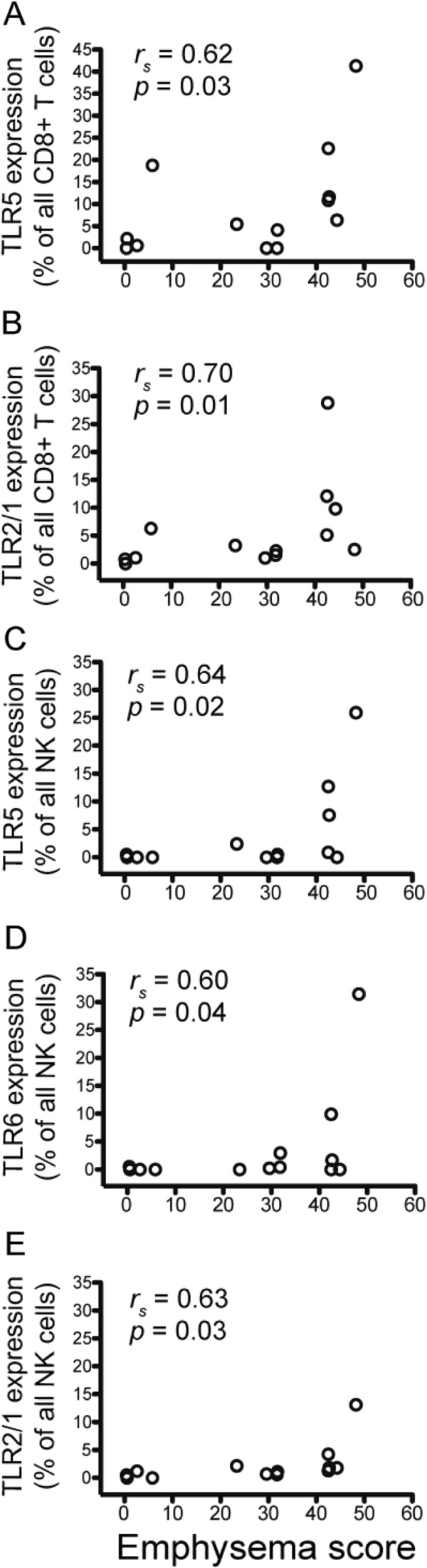
**Expression of TLR5 and TLR2/1 on lung CD8+ T cells and NK cells increases with worsening emphysema.** Lung tissue was stained with monoclonal antibodies to measure TLR expression on T cells, NK cells, and NKT cells. Expression (vertical axis) of (**A**) TLR5 on CD8+ T cells, (**B**) TLR2/1 on CD8+ T cells, (**C**) TLR5 on NK cells, (**D**) TLR6 on NK cells, (**E**) TLR2/1 on NK cells, stratified by percent emphysema (−950 HU threshold) (horizontal axis). Open circles represent individual patients, *n* = 13. Spearman non-parametric analysis was used to calculate the *r*_*S*_ value.

Expression of TLRs by lung CD8+ T cells also showed correlation with percent emphysema, calculated from non-contrast computed tomography (CT) scans that were available for 13 patients. The percentage of lung CD8+ T cells expressing TLR5 and TLR2/1 increased with worsening emphysema (Table 3 and Figure 4A,B). Similarly, NK cells expressing TLR5, TLR6, and TLR2/1 also correlated with worsening emphysema as determined by CT quantification (Table 3 and Figure 4C-E). The magnitude and direction of these correlations remained similar, although significance was attenuated after adjustment for age and smoking status as independent variables. The expression of TLRs on lung CD4+ T cells and NKT cells did not correlate with percent emphysema, implying that the regulation of TLRs within the lung environment in a given patient differs between lymphocyte subsets.

**Table 3:** Summary of correlation between percentage of lung subsets expressing TLRs and emphysema score

**Subset**	**TLR**	***r***_***S***_**Value**	***p*Value**
CD8+	TLR1	**0.54**	**0.09**
T Cells	TLR2	**0.59**	**0.06**
	TLR4	**0.37**	**0.23**
	**TLR5**	**0.62**	**0.03**
	TLR6	**0.59**	**0.05**
	**TLR2/1**	**0.70**	**0.01**
CD4+	TLR1	**0.09**	**0.77**
T cells	TLR2	**0.52**	**0.09**
	TLR4	**0.11**	**0.72**
	TLR5	**0.30**	**0.32**
	TLR6	**0.21**	**0.50**
	TLR2/1	**0.27**	**0.39**
CD56+	TLR1	**0.51**	**0.09**
NK cells	TLR2	**0.47**	**0.12**
	TLR4	**0.35**	**0.24**
	**TLR5**	**0.64**	**0.02**
	**TLR6**	**0.60**	**0.04**
	**TLR2/1**	**0.63**	**0.03**

### TLR stimulation of lung CD8+ T cells results in increased cytokine production

**Figure 5:**
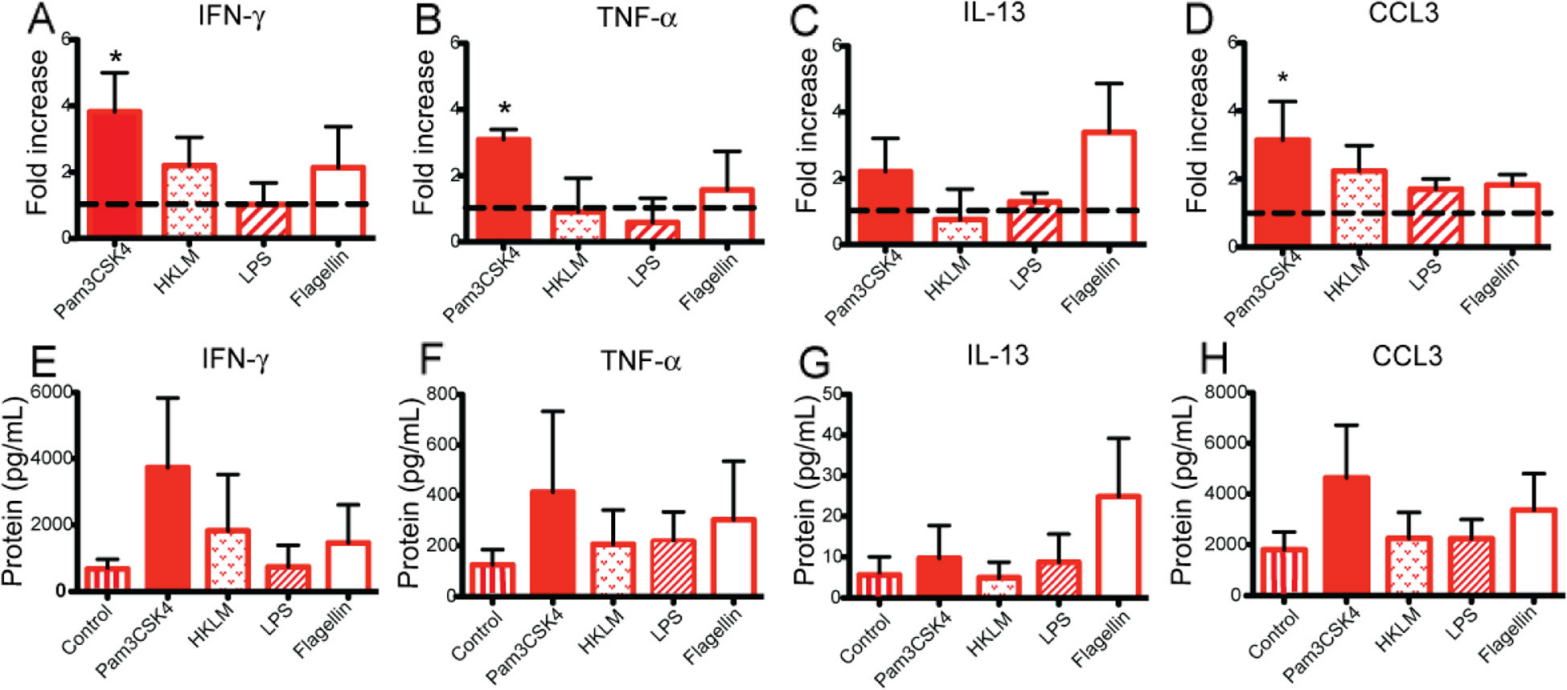
**Stimulation of TLR2/1 on lung CD8+ T cells increases production of IFN-γ TNF-α and CCL3.** CD8+ T cells were isolated from lung tissue of COPD patients (*n* = 7) and cultured for 48 hours with anti-CD3ε alone or with addition of specific ligands for the following TLRs (in parentheses): Pam3CSK4 (TLR2/1), HKLM (TLR2), LPS (TLR4), and Flagellin (TLR5). Supernatants were measured using multiplex beads for (**A, E**) IFN-γ, (**B, F**) TNF-α, (**C, G**) IL-13, and (**D, H**) CCL3. (**A-D**) Results are expressed as the fold change in protein concentration over the control stimulated with anti-CD3ε alone (represented by dashed line) or (**E-H**) as absolute values of protein. Bars represent the mean ± SEM. Kruskal-Wallis One-way ANOVA was used to look for differences between groups. *, p < 0.05, compared to the control.

Based on our previous finding that human lung CD8+ T cell transcripts for IFN-γ, perforin, and granzyme B are increased in later stages of COPD [6,7], we hypothesized that augmented production of these molecules might result from TLR-mediated response to danger signals in the lung environment. To test this hypothesis, CD8+ CD56- T cells isolated from lung tissue of COPD subjects were stimulated in vitro with plate-bound anti-CD3ε, plus each of the following TLR ligands: Pam3CSK4, heat-killed *Listeria monocytogenes (*HKLM), Poly(I:C), LPS, Flagellin, FSL-1, Imiquimod, ssRNA40, ODN 2006. Cells were stimulated for 48 hours and then supernatants were collected for protein analysis. Multiplex beads were used to measure IFN-γ, TNF-α, IL-13, perforin, granzyme A, granzyme B, and soluble fas ligand. Only Pam3CSK4, the ligand for TLR2/1, induced significant increases in IFN-γ and TNF-α (Figure 5A,B). Due to variability in baseline response between individual subjects, we first analyzed results expressed as the fold-change compared to the control stimulated with anti-CD3ε alone (*n* = 7). None of the other agonists induced significant increases in expression (HKLM, LPS, and Flagellin shown in Figure 5A-C; results not shown for Poly(I:C), FSL-1, Imiquimod, ssRNA40, or ODN 2006). Furthermore, the effect of TLR2/1 stimulation appeared limited to Tc1 cytokine expression, as there was no increase in either IL-13 (Figure 5C) or cytotoxic molecules (data not shown). Stimulation of the lung CD8+ T cells with the TLR2/1 agonist Pam3CSK4 also resulted in a 3-fold increase in the chemokine CCL3 (Figure 5D). No significant differences were observed in the other chemokines measured (CCL2, CCL4, CCL5, CCL11 and CXCL9). No increase in protein levels over the unstimulated control was seen when lung CD8+ T cells were cultured with TLR ligands in the absence of anti-CD3ε (data not shown), suggesting that TLRs function solely as co-stimulatory molecules, as has been reported, and do not independently activate lung T cells. Of the seven COPD subjects used in these in vitro experiments, three had lung resections and four were explants; six subjects were taking ICS. Thus, although surgical indication did not appear to play a role in the increase production of IFN-γ, TNF-α, and CCL3, we are unable to rule out a possible effect of the ICS. The absolute values for IFN-γ, TNF-α, IL-13, and CCL3 are also shown (Figure 5E-H). Following stimulation with anti-CD3ε and Pam3CSK4, IFN-γ and CCL3 averaged 4,000 pg/mL, which was 10-fold higher than TNF-α and 100-fold greater than IL-13 levels. Although there is an increase in the average of IFN-γ, TNF-α, and CCL3 following stimulation with Pam3CSK4, the data did not achieve statistical significance, due to the marked variation in baseline response between individual subjects.

**Figure 6:**
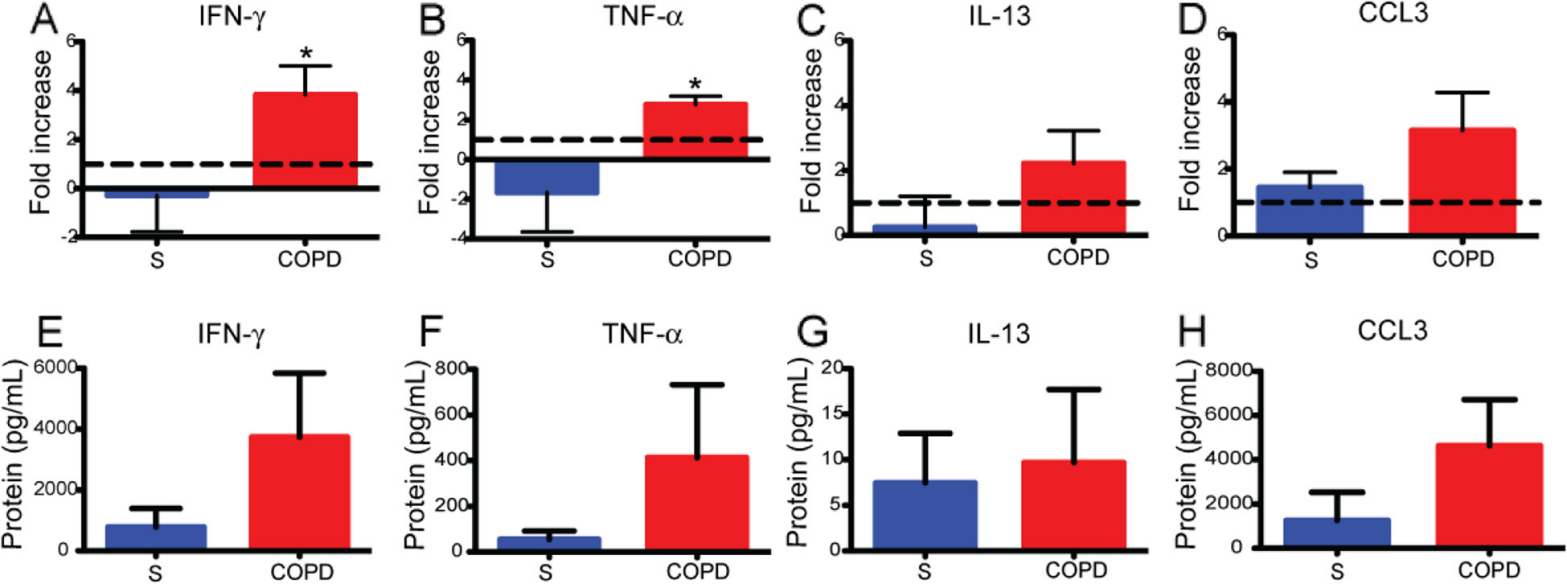
**Lung CD8+ T cells from COPD subjects have increased production of IFN-γ and TNF-α than CD8+ T cells from subjects without COPD. A-D**, CD8+ T cells were isolated from lung tissue of smokers without COPD (S, *n* = 5) for comparison to CD8+ T cells from subjects with COPD (*n* = 7) and cultured with anti-CD3ε plus Pam3CSK4 for 48 hours. Supernatants were measured using multiplex beads for (**A, E**) IFN-γ, (**B, F**) TNF-α, and (**C, G**) IL-13 and (**D, H**) CCL3. (**A-D**) Results are expressed as the fold change in protein concentration over the control stimulated with anti-CD3ε alone (represented by dashed line) or (**E-H**) as absolute values of protein. Bars represent the mean ± SEM. The Mann–Whitney t-test was used to compare control versus COPD subjects. *, p < 0.05, compared to the control.

Finally, to determine whether the increase in IFN-γ, TNF-α and CCL3 following TLR2/1 stimulation was specific to lung CD8+ T cells from COPD subjects, we also exposed lung CD8+ CD56- cells from control subjects to Pam3CSK4 (Figure 6A-D). Lung CD8+ T cells from COPD subjects (*n* = 7) produced significantly more IFN-γ and TNF-α than CD8+ T cells from control subjects (*n* = 5). No difference was seen in the production of IL-13 or CCL3. These results suggest that in COPD, the increased number of lung CD8+ T cells expressing TLR2/1 are able to contribute to an augmented inflammatory response following TLR2/1 stimulation. The absolute values for IFN-TNF-α, IL-13, and CCL3 are shown (Figure 6E-H).

## Discussion

The principal results of this analysis of TLR molecules on human lung T cells, NK cells and NKT cells demonstrate for the first time several observations relevant to COPD pathogenesis. First, expression of multiple TLRs on lung CD8+ T cells (but not on simultaneously analyzed lung CD4+ T cells, NK cells or NKT cells) was increased in COPD patients relative to smokers with preserved lung function. Second, TLR2/1 molecules expressed by lung CD8+ T cells were functional, as shown by the markedly enhanced production of cytokines linked to lung inflammation and COPD progression following co-stimulation by the TLR2/1 agonist in vitro. Third, expression by lung CD8+ T cells of TLR 2/1 and TLR5 correlated significantly with quantitative emphysema scores in univariate analysis. Collectively, these findings strengthen the evidence linking lung CD8+ T cells to smoking-induced lung destruction in susceptible cigarette smokers.

The finding that an increased percentage of lung CD8+ T cells express TLR1, TLR2, TLR2/1, TLR4 and TLR6 in COPD subjects extends to human lung parenchyma and to a specific disease state the results of previous studies of peripheral blood from healthy humans [16,19,20,26,27]. Although many TLRs have multiple ligands, it is noteworthy that increased expression was seen in the cell surface receptors most strongly associated with bacterial recognition (TLR1, TLR2, TLR2/1, TLR4 and TLR6), and not in the endosomal receptors TLR3 or TLR9, which recognize viruses and endogenous danger signals [28]. Furthermore, the very strong correlation between TLR5 or TLR2/1 and emphysema severity suggests that TLRs on lung CD8+ T cells may be specifically contributing to the pathogenesis of emphysema and highlights the importance of defining specific COPD phenotypes in selecting patients for personalized therapies [29]. Additional experiments will be needed to determine whether the co-stimulatory effect of TLR ligation is additive or synergistic when multiple TLRs are stimulated simultaneously, as would plausibly occur during exacerbations of COPD. Neither the presence of lung cancer nor recent infections appeared to contribute to the difference in TLR expression that we observed between healthy subjects and COPD subjects. Little is known about the effect of glucocorticoids on TLRs, but it has been shown that dexamethasone, in combination with IFN-γ or TNF-α, was able to synergistically enhance TLR2 expression on respiratory epithelial cells [30]; however in a separate study, dexamethasone was shown to downregulate TLR4 expression in an airway epithelial cell line [31]. The effect of steroids on TLRs has not been investigated in T cells. In our study, we saw no difference in TLR expression when ICS use was evaluated.

Although we also detected expression of multiple TLRs on lung CD4+ T cells, the near complete lack of their increased expression on this cell type in COPD, relative to age-matched smokers with preserved spirometry, implies either that the regulation or significance of TLR expression on CD4+ lung T cells, or the role of that subset itself, differs in COPD from that of lung CD8+ T cells. It is interesting that on the CD56+ NK cells, TLR5, TLR6, and TLR2/1 only showed a correlation with emphysema severity but showed no difference between COPD subjects and controls. These finding are compatible with the suggestion by Borchers and colleagues [32] that NK cells are particularly crucial in emphysema pathogenesis. Collectively, our findings support previous studies [33-37] on the importance of adaptive immune responses in emphysematous lung destruction. Whether TLR expression is restricted to specific CD8+ T cell clones, especially those with autoreactivity, will require further study, although the rather low fraction of total lung CD8+ expressing TLRs in the current study would be compatible with that possibility.

Lung CD8+ T cells in COPD appear especially sensitive to stimulation through the TLR2/1 heterodimer, as shown by the results of co-stimulation by the specific agonist Pam3CSK4. Our findings extend to the human system the finding that murine ovalbumin-specific OT-1 cytotoxic T lymphocytes responded to TLR2/1 stimulation with increased levels of IFN-γ at both the RNA and protein levels [38]. TLR2 is known to recognize the most diverse repertoire of microbe-associated patterns, in part through its unique ability to heterodimerize with either TLR1 or TLR6. TLR2/1 heterodimers are primarily responsible for recognizing triacylated lipoproteins, such as the outer membrane proteins of nontypeable *Haemophilus influenzae* (NTHI) [39]. This organism is one of the predominant bacterial pathogens associated with airway infection in COPD, both in stable disease and as an important infectious trigger of exacerbations [40,41]. Although the presence of TLR2/1 on lung CD8+ T cells might play a role in host defense in early COPD, repeated cycles of infection could swing the balance from host defense to inappropriate activation and subsequent tissue damage. Importantly, NTHI can be an intracellular pathogen of respiratory epithelial cells and macrophages [42,43], suggesting the possibility for presentation both of NTHI-derived antigens in the context of class I MHC molecules and of their lipoproteins to TLR2/1 on lung CD8+ T cells. A recent study by King and colleagues used live NTHI to stimulate T cells from the lungs of COPD subjects and control subjects [44]. They found that both CD4+ and CD8+ T cells from COPD subjects produced significantly higher levels of TNF-α, IL-17, and IL-13. Additional experiments will be needed to confirm whether TLRs have a role in this response.

The range of effector molecules significantly increased by co-stimulation via TLR2/1 (IFN-γ, TNF-α and the chemokine CCL3) is also intriguing. Transgenic overexpression of IFN-γ in the lungs induces production of matrix metalloproteinases by macrophages and development of emphysema in a murine model [45], Similarly, a central role for TNF-α in smoking-induced emphysema development is well-supported by data from murine models [46-48], likely reflecting in part the ability of TNF-α to activate endothelial cells to increase recruitment of inflammatory cells. CCL3 (previously known as MIP-1α) is a ligand for the chemokine receptors CCR1 and CCR5, and we previously showed that expression of CCR5 by lung CD8+ T cells increases with spirometrically-defined COPD severity [6]. Hence, stimulation via TLR2/1 and possibly other TLRs could generate a positive feed-back loop via CCR5 inducing the accumulation of lung CD8+ T cells seen in COPD.

Collectively, the current results agree with our previous finding that lung CD8+ T cells in COPD have a Tc1 phenotype and lack Tc2 cytokine secretion, even following TCR-dependent or TCR-independent stimulation [7]. The practical importance of the small, statistically insignificant increases in IL-13 protein production following stimulation with flagellin is questionable, because its concentration was so much lower than that of IFN-γ (100-fold less) and TNF-α (10-fold less). Because we had previously shown that perforin and granzyme B transcripts from lung CD8+ T cells were increased in severe COPD [7], we were surprised to see that TLR-stimulation did not increase production of perforin or granzyme B. It is possible that perforin and granzyme were increased in the intracellular granules of the CD8+ T cells but release of these molecules into the supernatant requires CD8+ degranulation. If the CD8+ T cells did not degranulate, then any increases in perforin or granzyme B would only be detectable with intracellular flow cytometry, which we did not perform in these particular experiments.

Our results differ in several respects from a previous study by Nadigel and colleagues, which found that both TLR4 and TLR9 were increased on lung CD8+ T cells from COPD patients [24], relative to healthy control subjects with normal spirometry who included two ex-smokers and three never-smokers. Although our study confirmed the finding of increased TLR4 expression, we did not see an increase in TLR9 expression in COPD relative to our reference population, who were entirely active smokers or ex-smokers. Furthermore, in our analysis, the percentage of lung CD8+ T cells expressing TLR4 only modestly increased in COPD, whereas they saw an increase from 20% in the control lung tissue to 90% in the COPD lung tissue [24]. These differences can likely be attributed to the tissue sample and techniques that were used. Nadigel et al. used immunofluorescence microscopy to analyze TLR expression of CD8+ T cells in endobronchial biopsies (i.e., relatively large airways) [24]. We used flow cytometry, which permits very objective quantification of specific staining relative to isotype control antibody, and we analyzed dispersed tissue from the distal lung compartment. CD8+ T cells are present in the central and peripheral airways and lung parenchyma in COPD, but the number of CD8+ T cells in the distal airways negatively correlates with airflow obstruction in patients with COPD [49], suggesting that this is a key location in COPD pathogenesis [3].

It is well documented that TLRs are expressed prominently on antigen-presenting cells, such as dendritic cells, and therefore play an important, if indirect, role in the initiation of adaptive T cell responses. TLR expression by macrophages and B cells can also contribute to production of antibodies and of chemokines. It is less clear whether the direct signaling of TLRs on T cells has the same physiological importance. On the one hand, the requirement for stimulation via the TCR, shown in this study and others, implies that TLRs are serving a primarily co-stimulatory role in CD8+ T cells. Indeed, numerous studies have shown that a number of TLR ligands can provide co-stimulatory signals to T cells, even in the absence of CD28 engagement [16-20]. Additional studies have suggested that TLR signaling through myeloid differentiation protein 88 (MyD88) may increase the clonal expansion and survival of activated T cells [50,51]. Conversely, the other lung cell types that express TLRs (dendritic cells, macrophages, B cells and epithelial cells) cannot produce IFN-γ, a unique cytokine that has both protective and potentially damaging properties. Thus, the current findings collectively suggest that lung CD8+ T cells use TLRs to sense and respond to microenvironmental conditions and to receive additional signals at the site of injury.

## Conclusions

In summary, human lung CD8+ T cells seem able to respond to danger signals in their environment, especially bacterial colonization of the lower respiratory tract, by up-regulating their potential to produce inflammatory mediators and cytotoxic molecules when stimulated via their cognate antigen receptors. Together with progressive upregulation of TLRs on lung CD8+ T cells as COPD worsens, this mechanism could contribute to lung destruction during respiratory infections.

## Abbreviations

CT: Computed tomography; COPD: Chronic obstructive pulmonary disease; FEV1: Forced expiratory volume in one second; FVC: Forced vital capacity; GOLD: Global Initiative for chronic Obstructive Lung Disease; HKLM: Heat-killed *Listeria monocytogenes*; ICS: Inhaled corticosteroids; NK: Natural killer; NTHI: Nontypeable *Haemophilus influenzae*; S: Smoker without COPD; TLR: Toll-like receptor.

## Competing interests

GRW, ALM, SWC, DAA, CAM, and LM have no competing interests to declare. F.J.M. has been a member of advisory boards for Actellion, Ikaria, Merck, Pearl, Pfizer, Jannsen, GlaxoSmithKline, Schering Plough, Novartis, Nycomed, Genzyme, Forest/Almirall, MedImmune, AstraZeneca, Potomac, Bayer, Elan, Talecris, and Roche. He has been on the speaker’s bureau for Forest/Almirall, Nycomed, Bayer, Boehringer Ingelheim, GlaxoSmithKline, France Foundation, MedEd, NACE, and AstraZeneca. He has also been a member of steering committees for studies supported by Altana/Nycomed, GlaxoSmithKline, Gilead, Actelion, Johnson/Johnson, Mpex, UCB, and the National Institutes of Health. He has been an investigator in trials supported by Boehringer Ingelheim and Actelion. M.K.H. has been a consultant for Boehringer Ingelheim, Pfizer, GlaxoSmithKline, MedImmune, Novartis, Grifols Therapeutics, and United Biosource Corporation. CMF and JLC are supported by research grants as outlined in the Endnotes.

## Authors’ contributions

CMF: Designed and performed experiments, analyzed data, produced graphs and tables, wrote manuscript; FJM: performed analyses and assisted in procuring human specimens, reviewed manuscript; MKH: performed analyses, reviewed manuscript; GRW: obtained emphysema scores from CT scans, reviewed manuscript; ALM: assisted in planning of experiments, reviewed manuscript; SWC: oversaw collection of surgical lung specimens, reviewed manuscript; DAA: aided in collection of surgical lung specimens, reviewed manuscript; CAM: consented surgical subjects and maintained records; LM: consented surgical subjects, maintained records, ensured compliance with IRB; JLC: secured research funding, oversaw study design, analyzed data and assisted in generating the final manuscript. All authors read and approved the final manuscript.
